# Real-Time Pedestrian Tracking Terminal Based on Adaptive Zero Velocity Update †

**DOI:** 10.3390/s21113808

**Published:** 2021-05-31

**Authors:** Ran Wei, Hongda Xu, Mingkun Yang, Xinguo Yu, Zhuoling Xiao, Bo Yan

**Affiliations:** School of Information and Communication Engineering, University of Electronic Science and Technology of China, Chengdu 611731, China; weiran_uestc@outlook.com (R.W.); xuhongda@std.uestc.edu.cn (H.X.); mingkunyang@std.uestc.edu.cn (M.Y.); xinguo_yu@outlook.com (X.Y.); yanboyu@uestc.edu.cn (B.Y.)

**Keywords:** zero velocity update, CNN, PYNQ, pedestrian dead reckoning, real-time terminal

## Abstract

In the field of pedestrian dead reckoning (PDR), the zero velocity update (ZUPT) method with an inertial measurement unit (IMU) is a mature technology to calibrate dead reckoning. However, due to the complex walking modes of different individuals, it is essential and challenging to determine the ZUPT conditions, which has a direct and significant influence on the tracking accuracy. In this research, we adopted an adaptive zero velocity update (AZUPT) method based on convolution neural networks to classify the ZUPT conditions. The AZUPT model was robust regardless of the different motion types of various individuals. AZUPT was then implemented on the Zynq-7000 SoC platform to work in real time to validate its computational efficiency and performance superiority. Extensive real-world experiments were conducted by 60 different individuals in three different scenarios. It was demonstrated that the proposed system could work equally well in different environments, making it portable for PDR to be widely performed in various real-world situations.

The work in this paper proposed a lightweight and real-time pedestrian tracking model for hardware acceleration and terminal implementation. The work enabled the implemented terminal to measure the real-time pedestrian trajectory in varying scenarios. Part of this work was published in the IEEE Global Communications Conference (GLOBECOM). The co-author Xinguo Yu in this paper holds the copyright to the published conference paper.

Conference paper information: Xinguo Yu, Ben Liu, Xinyue Lan, Zhuoling Xiao, Shuisheng Lin, Bo Yan and Liang Zhou. AZUPT: Adaptive Zero Velocity Update Based on Neural Networks for Pedestrian Tracking. In Proceedings of the 2019 IEEE Global Communications Conference (GLOBECOM), Waikoloa, HI, USA, 9–13 December 2019; pp. 1–6, doi:10.1109/GLOBECOM38437.2019.901407.

In that version, we proposed a method based on convolution neural networks (CNNs), which could adaptively pick ZUPT points from different motion types of different pedestrians. The current journal submission is a significant extension of the conference paper. The additional contributions of the journal submission were:

1. We improved the AZUPT model to make it sufficiently lightweight to be implemented in real time and applied an FPGA to accelerate the AZUPT algorithm, enabling real-time processing of pedestrian trajectory (Section 3);

2. We successfully designed the pedestrian tracking model with a reconfigurable neural network and implemented the model on Pynq-Z7 Soc, a lightweight platform to track trajectory in varying scenarios, allowing portability (Section 4);

3. Extensive additional experiments on pedestrian tracking involving more application scenarios and different motion types were conducted based on the terminal, to further demonstrate the accuracy and effectiveness of the proposed pedestrian-tracking model (Section 5);

4. The hardware performance including the energy consumption, computing resources, etc., were carefully evaluated on both a CPU and a GPU to demonstrate the efficiency and effectiveness of the proposed model (Section 5).

## 1. Introduction

There is great demand for precise navigation systems for pedestrians in both indoor and outdoor environments [1,2]. Currently, the Global Positioning System (GPS) cannot work in areas where satellites cannot transmit signals (underground or indoor environments), which leads to low efficiency of the GPS [3] in such environments. Therefore, most of the current positioning services are provided in outdoor environments [4]. However, the demand for indoor positioning is huge [5]. In terms of the transmission mode of positioning data, wireless positioning systems based on certain infrastructures are widely used. The available wireless communication media include RFID, Bluetooth, WiFi, ultrasonic, etc. [6]. However, these kinds of communication are susceptible to noise, so a large number of access nodes should be deployed in advance to avoid noise interference, which requires a huge workload.

Unlike infrastructure-dependent localization systems, pedestrian dead reckoning (PDR) using an inertial measurement unit (IMU) has been more stable for versatile tracking systems. The IMU consists of a triple-axis accelerometer and a triple-axis gyroscope, and these sensors enable the measurement of step length and heading direction [7,8]. However, drift exists in IMUs, and the accumulated errors make it hard to predict the real pedestrian trajectory. Zero velocity update (ZUPT) has been adopted as an effective approach to eliminate the system error [9,10]. The ZUPT implemented with the Kalman filter not only rectifies the velocity, the position, and the attitude errors, but also reduces the influence of the drift accumulation from the IMU [8,11].

**Figure 1:**
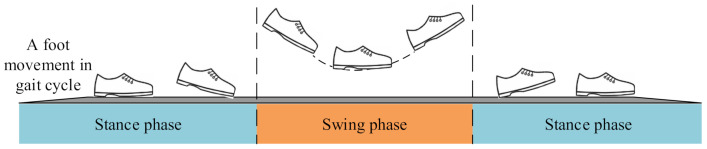
Pedestrian gait cycles.

The pedestrian gait cycle consists of two phases: the stance and swing phase (illustrated in Figure 1). In the ZUPT method, when the carrier (foot wearing the IMU) is in a static state, the speed is zero at this time (stance phase, where ZUPT points appear). The foot is in the motion state for a longer time (swing phase, where non-ZUPT points appear)  [8,12]. The stance phases (ZUPT points) of each gait cycle play an important role in the reckoning of the long-term trajectory, so they have to be detected accurately [13] (details shown on the first page). Most of the stance phase-detection algorithms are threshold based, which fail to perform reliably across a variety of gait motions. Therefore, the accurate classification of the zero velocity update points is particularly important. This paper adopted a deep neural network to solve this long-standing problem in ZUPT, which was the adaptive zero velocity update (AZUPT) [14] model, to determine moments when ZUPT should be conducted. In terms of different motion types and walking patterns, AZUPT can ensure nearly identical performance.

Deep neural networks contain a large number of mathematical operations. Therefore, the prediction of the trajectory takes a long time. To accelerate the ZUPT algorithm, this paper selected FPGA hardware as the implementation platform.

The implementation of the PDR [15,16] was performed on the PYNQ-Z2 demo board, which is an ARM + FPGA dual architecture platform. Its fast operation speed, low power consumption, and high portability made the validation of inertial navigation achievable. The main contributions of this paper were as follows:

- Adaptive ZUPT points’ selection: We proposed a method based on convolutional neural networks (CNNs), which could adaptively pick ZUPT points from different motion types of different pedestrians (e.g., walking, fast walking, and running);
- AZUPT terminal implementation: The whole tracking system including AZUPT was implemented on the Zynq-7000 SoC platform to validate its computation efficiency and performance superiority;
- Extensive real-world validation: Extensive experiments were conducted in multiple indoor and outdoor experiments by 60 different individuals in walking, fast walking, and running modes.

The remainder of this paper is organized as follows: Section 2 introduces the related work. Section 3 offers the details of the CNN-based ZUPT points’ selection method. Section 4 outlines the system architecture. Section 5 introduces the hardware implementation and result evaluation of the AZUPT algorithm based on the Zynq-7000 SoC platform. Section 6 presents our experimental results and the evaluation of the AZUPT algorithm. Section 7 concludes the paper and discusses ideas for future work.

## 2. Related Work

Strap-down inertial navigation systems (SINSs) and step-and-heading systems (SHSs) are the main approaches for PDR [17]. However, for both methods, the bias and noise of the IMU have a huge impact on the location accuracy due to the IMU’s small size and limited hardware performance [18]. Integration with signals of external sensors and ZUPT are two main methods to reduce error accumulation caused by bias and noise.

In past work, Ricardo Anacleto et al. used IMU and GPS data fusion, while the system could work only when the environment was well covered by satellite signals [19,20]. B. Kazemipur et al. proposed a vision-based system that used vision information to assist localization [21,22]. However, the vision-based system required a huge amount of calculation; and it was unstable, affected by the viewchanges in the environment. Z. Xiao-dong et al. combined the particle filter and indoor map information to realize a positioning system. Nevertheless, building maps are usually inconvenient to obtain in advance [23]. In some research, the magnetometer was set to evaluate the orientation [24], but magnetic fields are highly susceptible to environmental effects [25]. Thus, these systems mentioned above are defective and not widely used.

Researchers proposed the ZUPT method to reduce the trajectory drift by detecting the stance phase of each gait cycle [8], and the proper detection of zero velocity moment is the key point to ZUPT. In traditional approaches, the acceleration and angular velocity are analyzed and compared with the preset threshold to determine the stance phase [7,26]. Nevertheless, the threshold is closely related to the gait motions and features of different pedestrians [26], which means that a reasonable threshold is changeable. To overcome this problem, researchers proposed a dynamic threshold method to select thresholds according to different velocities [27,28]. However, the output is usually the range of threshold values, which is not precise and even results in an erroneous judgment of the stance phase.

As deep learning has developed rapidly in recent years, researchers have tried to apply it to PDR. Hannink et al. trained a deep CNN to map stride-specific inertial sensor data to the resulting stride length [29]. A team from Oxford University put forward a deep neural network (DNN) framework to set up the end-to-end model. This DNN model solved the problem of the long-term drift of inertial sensors [30]. Additionally, a team from University of Toronto Institute for Aerospace Studies took advantage of long short-term memory (LSTM) to explore robust inertial navigation [31], and they also improved the accuracy of the zero-velocity-aided inertial navigation system. However, the indoor location algorithm contained numerous operations, and the use of deep learning further increased the computation cost. Extensive calculations made it tough to predict the trajectory in real time.

Field-programmable gate arrays (FPGAs) are highly proven to accelerate algorithms due to the programmable gate’s high parallel computing capability. In the field of inertial navigation systems based on FPGAs, a team from Beijing University of Aeronautics and Astronautics used an FPGA as the data acquisition and storage module, which sent the data to the computer as the upper computer for processing [32]. Zhang Chunxi et al. designed a strap-down inertial navigation system (SINS) based on the system programmable chip (SOPC) with an FPGA [33]; the designed system realized the functions of data acquisition, error compensation, and navigation solution. However, none of these schemes accelerated the calculation speed of the navigation algorithm or related neural network algorithms with an FPGA.

FPGAs have been widely explored as hardware accelerators for neural network algorithms because of their high energy efficiency, computing capabilities, and reusability. Mittal, Sparshet al. surveyed techniques for implementing and optimizing CNN algorithms on FPGAs; they organized the works into several categories to bring out their similarities and differences [34]. In terms of large-scale CNNs, Naveen Suda et al. presented a system to maximize the throughput of an OpenCL-based FPGA accelerator (considering the FPGA resource constraints) for a given CNN model [35]. The application of the FPGA greatly sped up the deep neural network’s operation speed. However, the balance of the model performance and the resource usage of FPGAs largely depended on the designer.

## 3. CNN-Based ZUPT Points Selection Method

### 3.1. Dataset and Labels

In order to obtain a neural network model with high accuracy, a reliable dataset is vital. Besides, rich features from the training data can effectively overcome the problem of overfitting and can build a robust model.

**Sites and dataset:** To demonstrate the real-world applicability of the tracking system, our dataset was collected during three different motion types performed by sixty test subjects at seven different sites. The entire data-collecting process was carried out on flat ground. Overall, nine-hundred trajectories were traveled by the test subjects during the 40-day data collection, being approximately 1300 m long on average. The details are shown in Table 1.

**Participants:** To improve the generalization of the system, the variations among different people were taken into account by acquiring data from 60 people of a variety of genders, heights, weights, and ages. During the experiments, each of them was instructed to travel along all 15 routes performing one of three motion types consisting of (1) walking (W), (2) fast walking (FW), and (3) running (R), instead of taking a particular route performing a certain motion type. The specific information of the motion types of each trajectory is also shown in Table 1.

**Figure 2:**
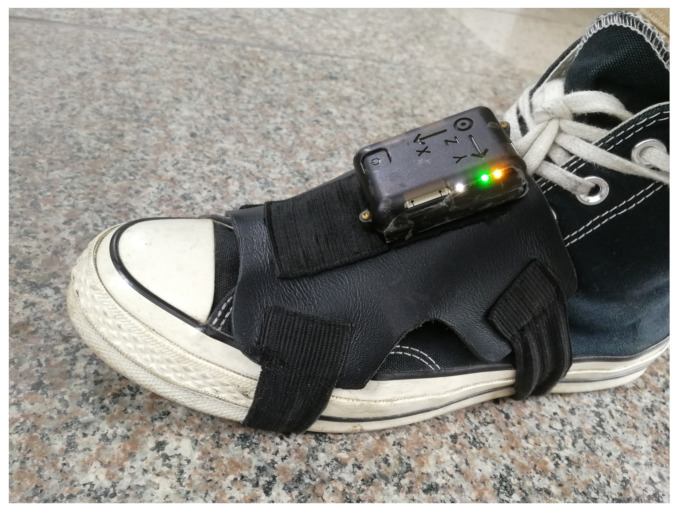
NGIMU attached on the left foot.

**Devices:** We used a next-generation IMU (NGIMU) including a triple-axis gyroscope, triple-axis accelerometer, and magnetometer. In our experiments, the IMU was attached on either foot, and we took it outside of the wrapping for demonstration, as shown in Figure 2. The information of each sensor on the NGIMU is summarized in Table 2.

**Threshold-based ZUPT points’ selection:** Before dataset construction and label generation, we introduce the fixed threshold ZUPT points’ selection method. After adjustment of the threshold, this threshold-based algorithm can preliminarily classify the ZUPT points, which comprises the basics for labeling.

**Figure 3:**
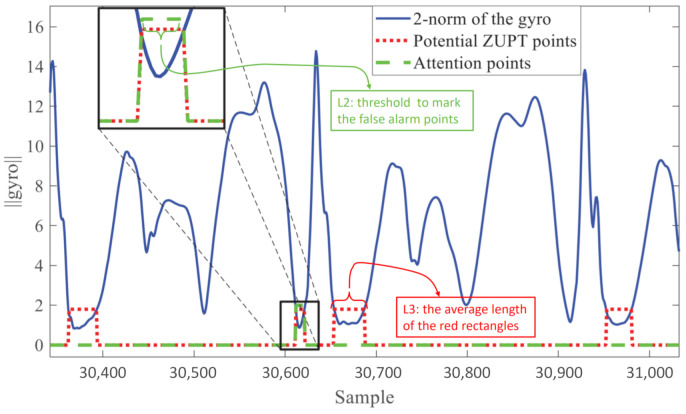
Two-norm value of the gyroscope data during a running sequence. Note that points encircled by small green rectangles (dashed lines) may be false alarm points, which would damage the trajectory, especially during the motion types of fast walking and running.

The stance phase of the gait cycle can be obtained from the data of the gyroscope or accelerometer. It can also be obtained from the linear combination of the data from both sensors. As shown in Figure 3, our proposed method took advantage of the fact that the two-norm value of gyroscope data was generally small during the zero-velocity period (relatively steady). In this paper, we set a certain fixed threshold value for zero-velocity-points’ detection as follows:

(1) $$\begin{array}{cc} \; & zv_{k}=\left\{\begin{array}{cc} ZUPT\;point, & ∥gyro_{k}^{b}∥\;<\;th_{gyro}\\ non-ZUPT\;point, & otherwise \end{array}\right. \end{array}$$

In this function, $∥gyro_{k}^{b}∥$ is the two-norm value of the gyroscope data at sampling step *k*. The superscript *b* denotes that the vector is in the sensor body coordinates. $∥gyro_{k}^{b}∥$ is calculated as:

(2) $$∥gyro_{k}^{b}∥=\sqrt{{\left(w_{x,k}^{b}\right)}^{2}+{\left(w_{y,k}^{b}\right)}^{2}+{\left(w_{z,k}^{b}\right)}^{2}}$$

*w* in Equation (2) denote the angular velocity values of the three-axis gyroscope in carrier *b* at the *k* sampling moment point ($w_{x,k}^{b}$, $w_{y,k}^{b}$, and $w_{z,k}^{b}$ correspond to the *x*-axis, *y*-axis, and *z*-axis, respectively).

$th_{gyro}$ is the fixed threshold value, and $zv_{k}$ shows that the point is a ZUPT point or a non-ZUPT point. This means that when the two-norm value of a point was less than the fixed threshold, the point was considered to be a ZUPT point. Otherwise, it was regarded as a non-ZUPT point.

**Generate the label:** As mentioned above, the two-norm value of the gyroscope data (directly acquired from IMU) changed periodically with the gait cycle, as shown in Figure 3. An optimal threshold of the two-norm value of the gyroscope data existed for each motion type of each participant. We considered the potential ZUPT points if their two-norm value of the gyroscope data was less than the fixed threshold (the optimal threshold fixed for each participant; we obtained the threshold for a certain individual by extensive iterative selections until the participant’s trajectory approximated the ground truth). Then, we marked the potential ZUPT points in red rectangles (dotted lines) and measured the average length of ZUPT points’ L3 (the average length of the red rectangles). Small burr signals (the signals that misjudged the non-ZUPT point as the ZUPT point) interfered with the fixed threshold selection method, so we built an algorithm to filter the burr signals and detect the ZUPT points; its details are shown the pseudocode as follows.

In Algorithm 1, L1 and L2 are presetconstants. Their values were adjusted according to the trajectory’s performance and needed to be selected many times until they were the appropriate values (we obtained the most suitable L1 and L2 by iteratively screening them until the pedestrian trajectory approximated the ground truth). Additionally, L1 must be a minimal value, and L2 was less than L3.

If the length of the marked potential ZUPT points was less than the predetermined small constant value L1, it proved that the sampling length contained extremely few points without the foot’s landing time, so the points in the sampling length could be judged as non-ZUPT points. If the length of potential ZUPT points was greater than L1, but less than the predefined empirical value L2 (L2 < L3), then it was hard to determine whether the corresponding sampling points were ZUPT points, so the algorithm would mark these points in green dashed rectangles. Finally, a manual review was performed on the points in green rectangles, and they were corrected if they were non-ZUPT points. We note that points encircled by a small green rectangle may be false alarm points that would damage the trajectory, especially during the motion types of fast walking and running.

### 3.2. CNN-Based ZUPT Points’ Selection Model

**Figure 4:**
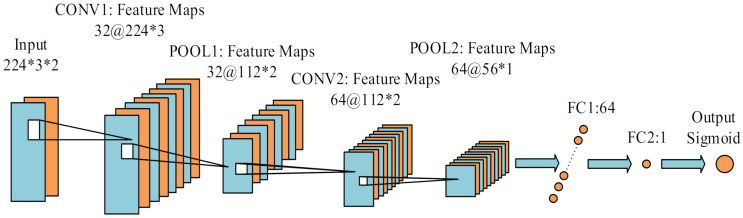
Structure of the ZUPT point selection model. In order to extract features from the last convolutional layer, the numbers of the first and second convolutional kernels are 32 and 64, respectively.

Figure 4 shows a schematic diagram of the ZUPT point selection model proposed in this paper.**Algorithm 1** ZUPT points’ selection algorithm.**Require:**  Gyroscope 2-norm value  Small constants L1 and L2; L1 is less than L2.**Ensure:**  ZUPT points and non-ZUPT points1:**for**k=0 to datasize **do**2:  **if** $∥gyro_{k}^{b}∥<Threshold$ **then**3:   Mark the points in red rectangles.4:   **if** length<L1 **then**5:    Remove red marks.6:   **else if** length<L2 **then**7:    Mark the points in green rectangles.8:   **end if**9:  **end if**10:**end for**


Our CNN model was mainly inspired by the philosophy of AlexNet [36], which is the most concise and classic image feature extraction network. Our dataset with only pedestrian’s velocities and accelerations contained fewer features, so we censored the network. The main body of the network was the convolutional layer and the pooling layer. The convolutional layers mostly had 3 × 3 filters with zero padding. We performed downsampling directly by convolutional layers that had a stride of two. The network ended with two fully connected layers with the sigmoid. The total number of weighted layers is four (two convolutional layers and two fully connected layers) in Figure 4.

The details of our CNN-based model are as follows: (1) An input layer with the pre-processed standard IMU measurements fed in. Input layer size: 224 × 3 × 2: 224 means we selected 224 time points for one sample to judge if the 113thpoint was a ZUPT point; 3 means we had 3-dimensional data for each point (*x*, *y*, *z* axis); and 2 indicates the observed velocities and accelerations of each point (corresponding to the orange and blue feature maps in Figure 4). (2) The first convolutional layer filtered the 224 × 3 × 2 input image with 32 kernels of size 3 × 3 with a stride of 1 pixel (this was the distance between the receptive field centers of neighboring neurons in a kernel map) (224 × 3 × 2 –> 224 × 3 × 32). (3) A max-pooling layer was set directly after the first convolutional layer, with the function to reduce the extracted features (224 × 3 × 32 –> 112 × 2 × 32), improving the computing speed and enhancing the model’s robustness. (4) The same as Steps (2) and (3), the second convolutional layer with a max-pooling layer screened the output from the last max-pooling layer for further feature extraction (112 × 2 × 32 –> 56 × 1 × 64). (5) Two fully connected layers extracted and combined the features of the last max-pooling layer (56 × 1 × 64 –> 64 –> 2). (6) An output layer of the sigmoid function based on the feature map from the last layer transferred the data to the output.

The accuracy of the framework obtained from the extensive training and test sets was 0.998 and 0.992, respectively. Since we only utilized and improved the fixed threshold method to label the data, the method did not influence the deep learning approach we used even if the threshold was related to pedestrians’ features and motion types.

The CNN-based model gave a new and precise zero velocity update point selection method. However, to achieve the whole PDR device, we still needed the other algorithm and modules to format our lightweight terminal.

## 4. System Architecture

In this section, the algorithm and hardware of our proposed real-time PDR terminal are shown in detail.

### 4.1. The Architecture of Algorithm

**Figure 5:**
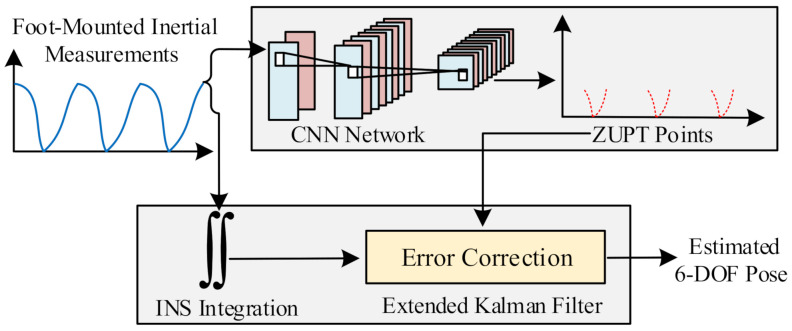
Block diagram of the AZUPT PDR system. Given any one of the motion types (walking, fast walking, running), AZUPT can pick ZUPT points accurately without recognition.

Figure 5 outlines the architecture of our positioning algorithm, which incorporated the CNN-based zero velocity detector. Firstly, the inertial measurements were fed into the CNN and the expanded Kalman filter, respectively. The CNN network was responsible for detecting the ZUPT points. Compared with the conventional fixed threshold method, the CNN solved the problem that the fixed threshold could not accurately classify ZUPT points due to the variation among individuals and motions. The CNN-based method, leveraging deep learning, realized the detection of the zero velocity adaptively. Combined with the calculated ZUPT points from the CNN, the extended Kalman filter (EKF) selected the zero-velocity as the pseudo-measurement (EKF states: attitude, velocity, and position). The zero-velocity measurements were fused with a dead reckoning motion model in the extended Kalman filter to significantly reduce error growth over time. Therefore, the zero velocity intervals could be classified precisely.

### 4.2. The Architecture of the Hardware

**Figure 6:**
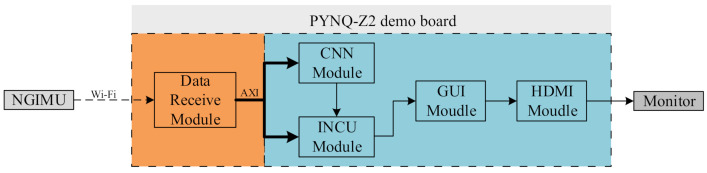
System architecture of the real-time PDR terminal system.

The overall structure of the lightweight PDR terminal system is shown in Figure 6, which included five modules: data receiving, CNN, INCU, GUI plotting, and HDMI control.

**Data-receiving module:** The data-receiving module collected acceleration and angular velocity data from the NGIMU module introduced in Section 3 at a sampling rate of 400 Hz, then this module packed the data into OSC (Open Sound Control) format and transmitted them to the processing system (PS) over WiFi. Considering that Xilinx officially provides Python library support for the PYNQ platform, the PS can quickly call the OSC Python library to complete the parsing of data packets, then send the parsed data to the INCU module and CNN module on programmable logic (PL) through the Advanced eXtensible Interface (AXI) bus.

**CNN module:** This module was built on top of the CNN network trained on TensorFlow, which produced a binary output (one or zero). Considering that pedestrians’ feet stayed on the ground for approximately 0.5 s and the sampling frequency of the NGIMU was 400 Hz, the input data sample was set to 224 points. For new data obtained by the CNN module, the sliding window with a sample length of 224 was shifted to contain the new information. Since the convolution layer needed a large amount of computation, we adopted the following two optimization strategies:

(a) parallel processing: multiple multiply-add operations performed in parallel to increase the operation speed [37,38];

(b) pipeline: multi-stage pipeline processing of data to make full use of DSP resources [39,40].

**Figure 7:**
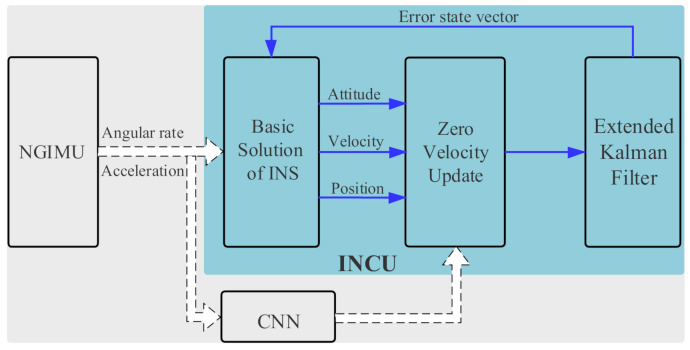
Block diagram of the INCU module.

**Inertial navigation computation and update (INCU) module:** As shown in Figure 7, firstly, low-pass filtering was conducted on the original data, which reduced the interference of noise. The pre-processed acceleration and angular velocity were then calculated to obtain the attitude, velocity, and position. The INCU module obtained the ZUPT point’s judgment result from the CNN module and then decided whether ZUPT was carried out after the calculation. Simultaneously, combined with the error EKF, the error state vector was obtained to update the attitude, velocity, and position.

**GUI plotting module and HDMI control module:** The GUI plotting module quantified the new pedestrian position obtained in the coordinate point. Then, combined with the collected real-time coordinate points, the GUI module completed the drawing of the pedestrian trajectory. The HDMI control module drove the monitor to enable the real-time update and display of the pedestrian trajectory.

## 5. Real-Time Pedestrian Dead Reckoning Implementation Based on the SoC Platform

In the last section, the architecture of the pedestrian dead reckoning system was described. In this section, we show the details about the implementation of the hardware architecture. Firstly, we picked a portable system on a chip (SoC) platform (PYNQ-Z2) as the realized terminal. Then, we implemented the proposed location system on the platform, with the real-time pedestrian trajectory displayed.

### 5.1. Hardware Platform

**Figure 8:**
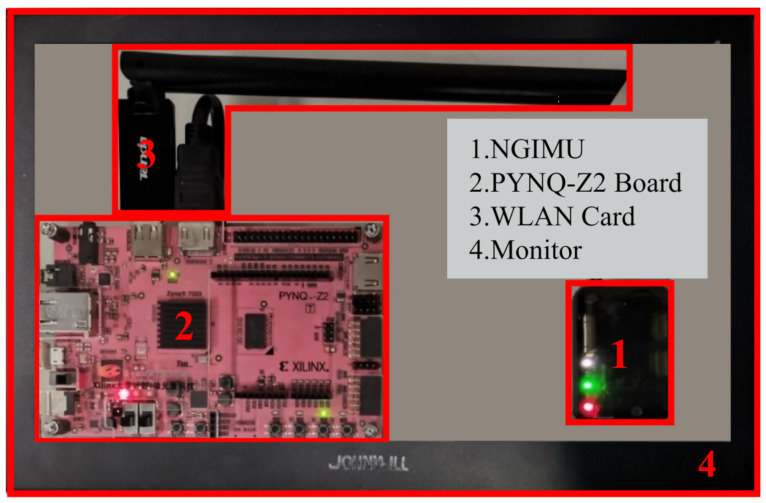
Hardware platform.

This subsection introduces the real-time PDR terminal’s four parts (shown in Figure 8): the NGIMU module, PYNQ-Z2 demo board, wireless network card, and portable monitor.

**Figure 9:**
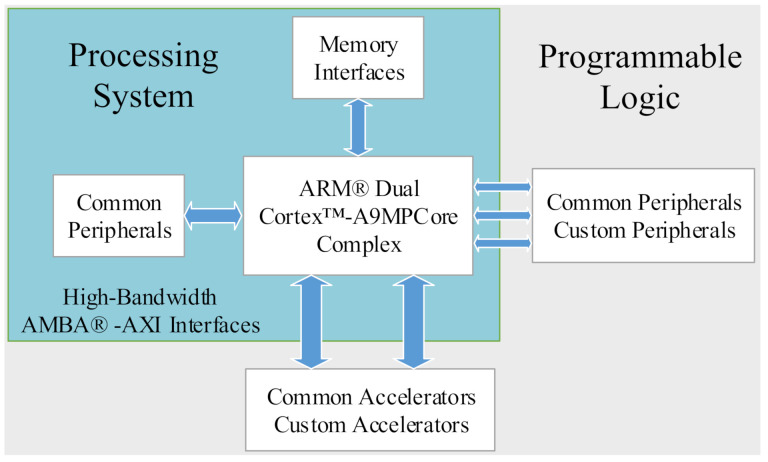
The internal structure of the XC7Z020.

The MEMS NGIMU is quite small, lightweight, and portable. However, the PDR system implemented on the CPU and the GPU (the PC’s integrated CPU and GPU; the PDR system was preliminarily tested on them) was bulky and had high power consumption. It could not meet the requirements of the low power consumption, low cost, and embeddability of IoT terminal equipment [14], limiting the widespread use of the PDR system. Considering the low power consumption of the FPGA and the flexible controllability of the ARM, we chose the PYNQ-Z2 demo board (Xilinx Zynq All Programmable SoC board) due to the advantages of both the FPGA and ARM to adapt to the design of the real-time PDR system. The integrated FPGA demo board was portable enough, as pedestrians could easily carry it on their backs. The core of PYNQ-Z2 is the XC7Z020 chip of the Zynq-7000 series, and its internal structure is shown in Figure 9.

XC7Z020 integrates two parts in a single chip: processing system (PS) based on the ARM’s dual-core Cortex-A9 processor and Programmable Logic (PL) based on the Xilinx programmable logic system, namely the FPGA. Firstly, the received data from the NGIMU module were temporarily stored in the PS through WiFi, then the PS transmitted the data to the PL through the internal high-speed Advanced eXtensible Interface (AXI) bus. The PL mainly carried out the data processing, including CNN network construction, inertial navigation computation and update (INCU), motion trajectory plotting, and High-Definition Multimedia Interface (HDMI) construction for the monitor.

### 5.2. System Implementation

**Figure 10:**
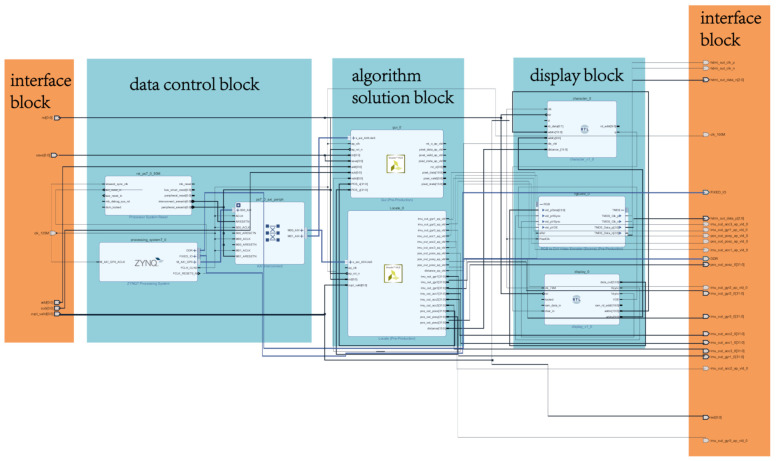
The whole block design of the system.

**Figure 11:**
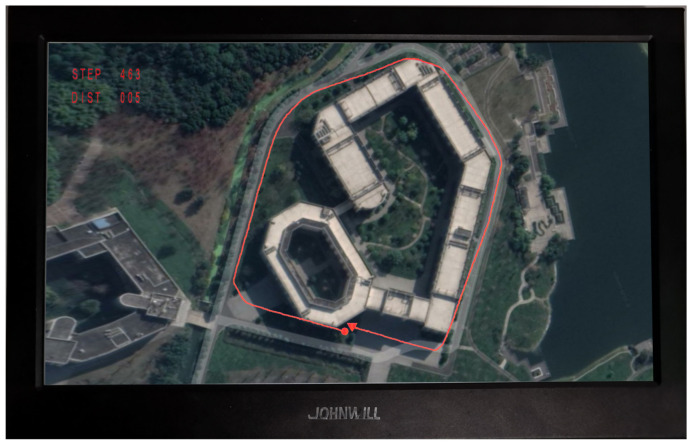
Effect of a complete running trajectory by the PDR terminal. The small triangle and the circle represent the starting point and the endpoint, respectively. STEP indicates the number of steps the test subject walked (the length of a STEP is the distance that the person moves when his/her left and right feet move once). DIST indicates the distance between the person’s current position and the starting point. Note that we chose to test on the running trajectory, which is the most difficult to predict among the three motion types, to prove the reliability of our PDR terminal.

The whole block design for the terminal is shown in Figure 10. All the modules described in Section 4 were implemented in IP cores (blue frames illustrated in Figure 11). The entire block was very complex, with dense route and data path tagging (note: these are not the purpose of Figure 11, so their letters are not distinct and can be ignored); it was simplified into four parts: interface, data control, algorithm solution, and display. This hardware process started with the data collected by the IMU module sent to the input interface block, and then, all blocks ran fast with intensive data transmission. The processed data were delivered to the display block, finally, to generate the trajectory output.

A large number of matrix operations were involved in the algorithm solution block. The operation of the display block was also extensive. Therefore, it was painful and laborious to describe the register-transfer level (RTL) directly using Verilog HDL, whereas it was easier to use the C language to implement it. High-Level-Synthesis (HLS), a useful high-level synthesis tool, supports the programming of Xilinx FPGA devices by the C, C++, and System C languages [16]. We used the HLS tools for development since they can quickly generate the intellectual property (IP) core of RTL [36] to improve development efficiency. Due to the HDMI driver’s rigorous time requirement, the HDMI control module was implemented using the Verilog HDL language, and this module can accurately describe the timing logic circuit.

The resource utilization of INCU, CNN, and GUI modules is shown in Table 3. The FPGA’s working frequency was 200 MHz, and the sampling frequency of the NGIMU module was 400 Hz, so the calculation period of a single sample must be less than 500,000 clock cycles. The implemented terminal (the running period of a data sample was about 140,000 clock cycles) could meet the calculation needs. The high-speed operating terminal could also ideally enable real-time processing and display the pedestrian trajectory.

**Figure 12:**
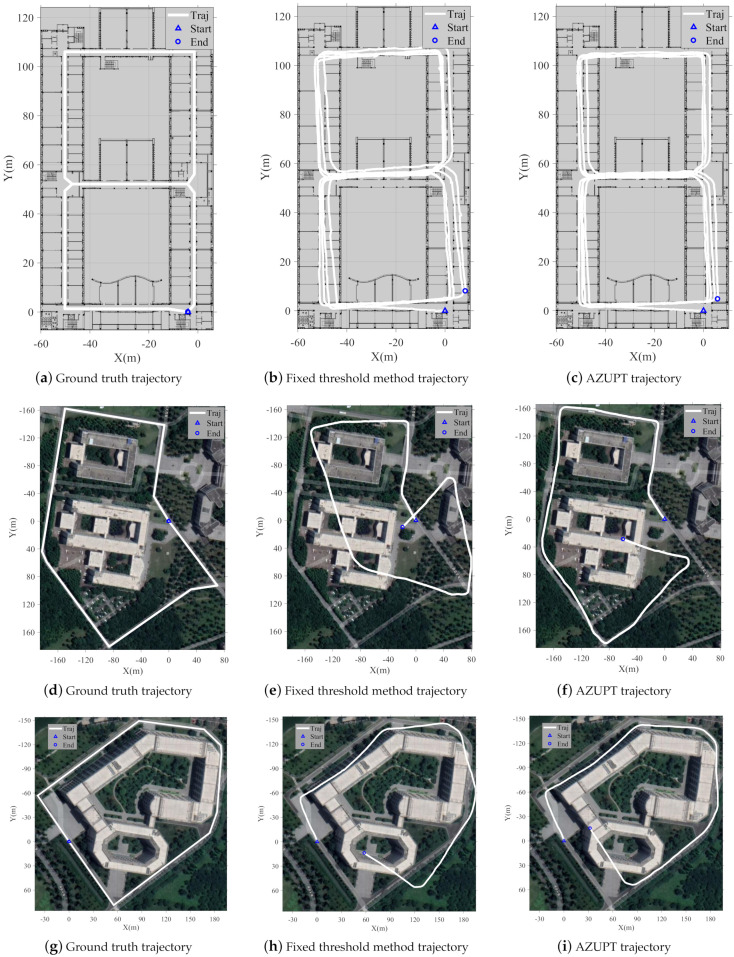
Experiments in the experiment building (top, walking), the scientific research building (middle, fast walking), and the first teaching building (bottom, running), showing the ground truth, fixed threshold method, and AZUPT trajectories. The fixed threshold method had a different result for different motion types and performed badly. However, the AZUPT method consistently outperformed the fixed threshold method and fit well with any of three different motion types.

To make the real-time trajectory accessible to the pedestrians (wearing the AZUPT hardware) themselves, we used a tiny monitor (shown in Figure 11) to project the GUI trajectory and perform the field implementation. The test subjects ran around the periphery of the first teaching building and returned to the starting point. The test result was shown on the screen in Figure 11, corresponding to the ground truth of Figure 12g. STEP indicates the number of steps the test subject walked (the length of a STEP is the distance that the person moves when his/her left and right feet both move once), and DIST indicates the distance between the person’s current position and the starting point. As shown in Figure 11, the pedestrian with the sensor walked 463 steps. The distance between the starting point and the endpoint was only 5 m, while the length of the whole track was 650 m, and the error rate (the distance between the starting point and the endpoint/the length of the whole track) was only 7.7%. Therefore, we realized the real-time plotting of the pedestrian trajectory on the SoC platform, which met the basic design requirements.

Through the test of the hardware platform, the effect of real-time processing on the SoC could be achieved by the proposed algorithm, which proved the algorithm’s effectiveness. Moreover, the algorithm could be easily converted to lightweight embedded devices, which also would achieve an excellent positioning effect.

## 6. Experiment

To visualize the performance of our AZUPT model, we used the lightweight terminal, as shown in Figure 11, to display the real-time trails combined with the map of a suitable size. To make the experiment persuasive, sixty different individuals participated in the test for three common motion types (i.e., walking, fast walking, and running), and all routes were long, closed loops.

### 6.1. Classification Accuracy

The proposed CNN-based zero-velocity detection algorithm performed well in selecting ZUPT points. Table 4 shows that the precision, recall, and F1-score (harmonic mean of precision and recall, better to evaluate the performance of the model) of the three motions were higher than 99%. Compared with the traditional fixed threshold method, our dynamic threshold method on the lightweight terminal provided a significant improvement.

### 6.2. Comparisons of the Three Motion Types

To demonstrate the applicability and robustness of the system in multiple scenarios, the AZUPT system was evaluated and compared against the fixed threshold approach in three real-world settings, namely the experiment building, scientific research building, and first teaching building. For the first trajectory, subjects with the foot-mounted IMU walked along the corridor four times, and the 440 m route for each time was shaped similar to an Arabic numeral eight.

Figure 12a,d,g shows the ground truth regarded as three experimental sites that corresponded to three kinds of common motion types in our daily lives. The known limitations of the NGIMU were the initial drift and low sensitivity. Without the selection of the proper ZUPT points, the estimated trajectory would drift and diverge seriously. With the conventional zero-velocity detection, namely the fixed threshold method, the tracks of the ZUPT-aided PDR algorithm are shown in Figure 12b,e,h. The fixed threshold performed well in walking, as shown in Figure 12b, because an optimal threshold might fit the slow-speed motion perfectly in spite of the pedestrian changing. However, in the case of fast walking and running, the trajectory obviously deviated, as shown in Figure 12e,h. The fast or large-amplitude swing of the feet (when pedestrians were running, their leg movements were more intense) could cause the appearance of false alarm points, which are encircled by small green rectangles in Figure 3. The method proposed in this paper solved the above problem by utilizing a neural network to adaptively select ZUPT points, which are illustrated in Figure 12c,f,i. In the labels’ selection process, false alarm points were removed, and missing points were artificially added to ensure the correct implementation of the ZUPT points’ selection.

### 6.3. The Cumulative Distribution Function of the Error

**Figure 13:**
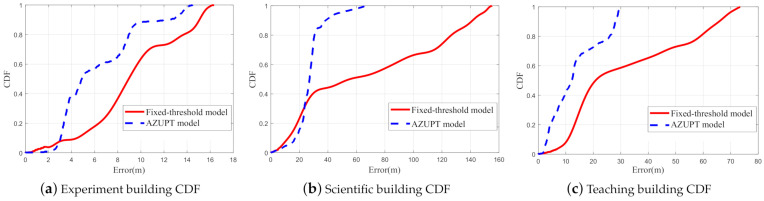
The CDF of the location errors with the fixed threshold model and the AZUPT model at different sites. Note that there were few false alarm points in walking for our AZUPT model to correct; therefore, our AZUPT method only showed relatively slight improvements compared to the fixed threshold method in Figure 7a.

Each subfigure in Figure 13 shows the cumulative distribution function (CDF) of the location errors in the experiment corresponding to one of three kinds of motion types and sites. We can see that the errors of the algorithm proposed in this paper were smaller than the corresponding errors of the fixed threshold method, which were calculated from the second column of Figure 12. Moreover, the CDF figures in Figure 13 matched well with Figure 12, which confirmed that our proposed method could operate well for the three motion types. Note that as we mentioned above, only via rough analysis, our proposed method did not show huge improvements compared to the conventional fixed threshold method for walking. The reason was that there were few false alarm points in walking, so that they did not have a remarkable effect on the estimation of the trajectory. In fact, our proposed method worked almost without any mistakes for the different motion types.

### 6.4. Platform Performance Analysis

Since the hardware structure and appropriate size of different acceleration devices were different, only comparing the calculation speed or throughput cannot measure the performance indicators. Therefore, this paper mainly compared the energy efficiency (*EE*) and energy efficiency ratio (*EER*) of each experiment group. The formula of *EE* is shown as follows:

(3) $$\mathrm{EE}=\frac{n}{E}=\frac{n}{P*t}=\frac{1}{P*\bar{t}}$$

In the formula above, *EE* represents the number of processed trajectory data points corresponding to per Joule of energy (the dimension is points/J), which can better reflect the experiment’s implementation efficiency. N is the number of calculated inertial navigation data points. *E* represents energy consumption. *P* is the platform power. *t* is the total calculation time. The over-lined *t* represents the average calculation time of each point. The power of the *FPGA* was obtained by analyzing the Vivado software platform provided by Xilinx. The *CPU* and *GPU* used rated power.

*EER* is the energy efficiency ratio of the two platforms. The corresponding expressions of *INCU* and *CNN* are shown in Equations (4) and (5), respectively:

(4) $${\mathrm{EER}}_{\mathrm{INCU}}=\frac{{\mathrm{EE}}_{\mathrm{SoC}-\mathrm{INCU}}}{{\mathrm{EE}}_{\mathrm{CPU}}}$$

(5) $${\mathrm{EER}}_{CNN}=\frac{EE_{\mathrm{SoC}-CNN}}{EE_{GPU}}$$

Based on the previously collected 900 sets of data, the *INCU* algorithm and *CNN* algorithm were implemented on the *CPU* and the *GPU*, respectively. Nevertheless, the system was large and not portable with a high power consumption, which constrained industrial PDR production promotion. To further promote the commercialization, we implemented these two modules simultaneously on the low-cost and small-sized SoC platform integrated with the FPGA. To obtain the performance indicators implemented on the SoC platform, the algorithms were tested on the *CPU*, *GPU*, and SoC platforms, respectively, and the test results (shown in Table 4) were analyzed.

As shown in Table 5, the Soc demo board was the PYNQ-Z2, and the core of the PYNQ-Z2 is a chip named XC7Z020CLG400-1 (a rudimentary chip, with minimal FPGA resources; only a few parallel processing processes can be performed). This disadvantage made the SoC inferior compared to the CPU and the GPU with respect to the INCU and CNN algorithms’ computing speeds. However, the PDR terminal could still meet the needs of real-time processing of data and plotting trajectories. Furthermore, in terms of the EER, SoC performed better, 12.58 times and 5.42 times better than the CPU platform and GPU platform.

### 6.5. Overall Terminal Assessment

Resource usage and chip power are essential evaluation indicators in algorithm implementations.

The resource utilization of the whole design was appropriate for the PYNQ-Z2 board’s capacity, as Table 6 illustrates. There were 208 slices of DSP (95%), 65 BRAMs (46%), 47,791 FFs (45%), and 49,091 LUTs (92%) used in the whole project (average resource usage ratio: 44.20%). The PYNQ-Z2 demo board with few resources and low cost met the requirements and had extra resources remaining.

**Figure 14:**
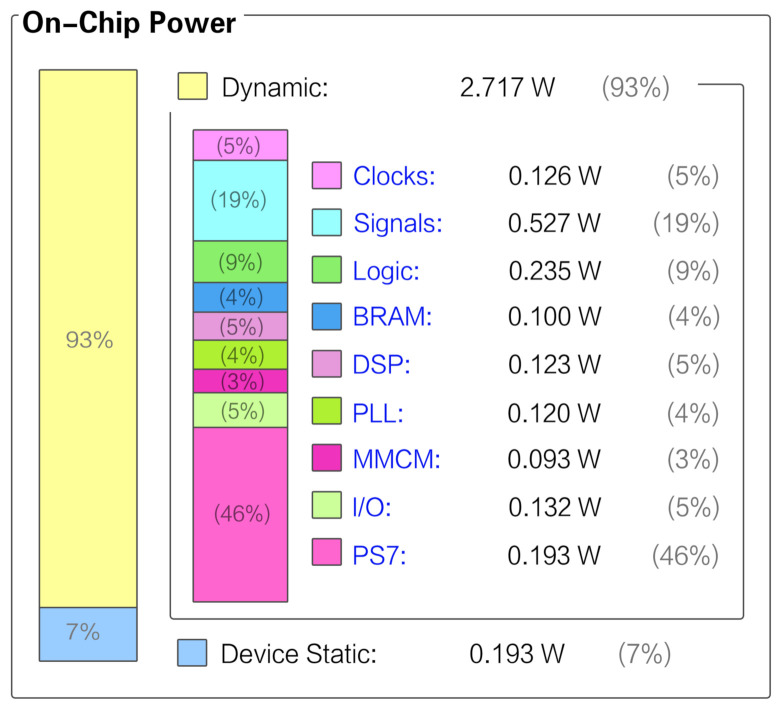
Power analysis from the implemented system.

The entire system worked with a low power consumption performance, only 2.91 W for the on-chip power, as Figure 14 shows. The total consumption consisted of two parts, in which the dynamic part shared a bigger proportion with 93%. In detail, forty-six percent of the power consumption was from PS7 (ZYNQ7 processing system), which was the core of the hardware. It controlled all modules’ coordinated operation and accurately made the data path, so the core output relative high power to guarantee the total system’s normal operation. However, our system’s total power consumption was far below the average navigation devices on the market. Considering the low-cost board we used, it was obvious that the PYNQ-Z2 platform could achieve a good balance among cost, power consumption, and computing ability.

**Figure 15:**
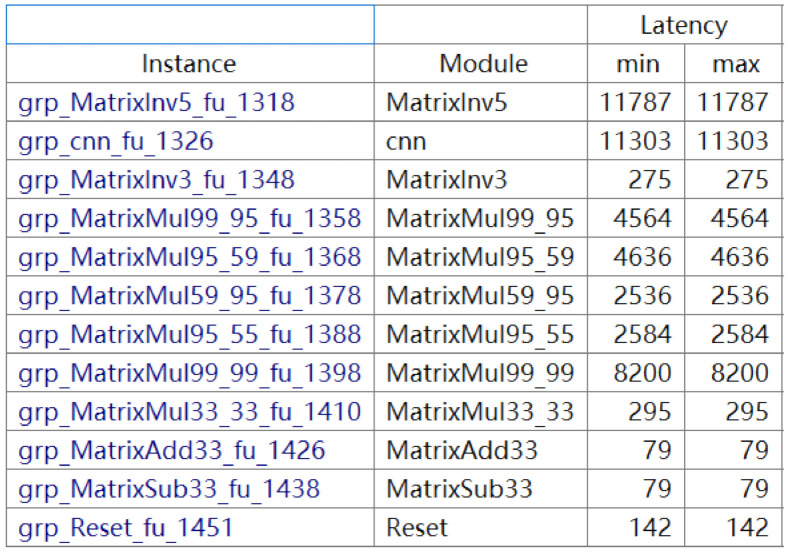
Latency of the modules from the HLS simulation.

Figure 15 illustrates the latency of each module in the proposed terminal; the unit for the latency is the clock cycle (the PYNQ-Z2 board’s working clock frequency is 200 MHz). The total algorithm latency of the hardware implementation was 66,697 clock cycles (about 0.3 ns), which meant it took only 0.3 ns to process the data (a set of triple-axis accelerations and angular velocities) once. As the tester moved, the algorithm drew a synchronized trajectory after hardware acceleration. Additionally, the process to determine the ZUPT points by CNN could be run synchronously with the plotting of the trajectory if the plotting started after a tiny time quantum, for example 1 s (the IMU’s frequency of sample extraction was 1.786 Hz). As a result, real-time tracking could be achievable.

## 7. Conclusions

In this paper, we adopted a method based on CNN that could adaptively pick ZUPT points regardless of the pedestrian types and motion types.

The AZUPT method was shown to outperform the traditional method in terms of both robustness and accuracy. Based on the AZUPT model, we validated the effectiveness of our system to estimate the trajectories that were more consistent with the ground truth, since its ZUPT classification accuracy was higher than 99% (shown in Figure 12c,f,i and Table 4). Both the fixed threshold method’s and the AZUPT method’s cumulative distribution function (CDF) of the location errors ensuredthat AZUPT generated less cumulative errors as pedestrians traveled (shown in Figure 13).

We also implemented a real-time PDR hardware on the Zynq-7000 SoC platform, making it capable of being widely used in various real-world applications. To evaluate the performance of the platform, we tested its chip power (only 2.717 W) and resource usage (average resource usage ratio: 44.20%) and also compared the energy efficiency of the terminal with other devices (Section 6.4). The result showed that the proposed terminal had the advantages of low power consumption and portability, which made real-time tracking achievable.

In the future, the tracking accuracy can be further improved by fusing a digital compass or millimeter-wave radar into the PDR-based autonomous positioning system.

## Figures and Tables

**Table 1:** Details of routes and motion types.

Site	Environment	Length	Motion Type
Experiment building	indoor	1760 (m)	Walking (W), FW
First teaching building	indoor	7,001,000 (m)	W, fast walking (FW)
Second teaching building	outdoor	1500 (m)	W, FW
Scientific research building	outdoor	1100 (m)	W, FW
First teaching building	outdoor	650 (m)	Running (R)
Athletics field	outdoor	800,720,550 (m)	R
A circle around campus	outdoor	4200 (m)	W

**Table 2:** Performance of sensors on the NGIMU.

Indices	Accelerometer	Gyroscope	Magnetometer
Dynamic Range	±16 g	±40,000 rad/h	±1300 T
Resolution	490 g	1.2 rad/h	∼0.3 T
Sample Rate	400 Hz	400 Hz	∼20 Hz

**Table 3:** Hardware module resource utilization.

Module	Resource Utilization			
	DSP	BRAM	FF	LUT
INCU	100	10	15,703	15,895
CNN	40	18	8713	13,025
GUI	25	8	7007	7183
Utilization Ratio	75%	46%	40%	81%
(Sum/Total)	(165/220)	(65/140)	(42,732/106,400)	(43,342/53,200)

**Table 4:** Detailed accuracy by the three different motion types.

Motion Types	Walk				Fast Walk				Run			
	Precision	Recall	F1-Score	Support	Precision	Recall	F1-Score	Support	Precision	Recall	F1-Score	Support
Non-ZUPT Points	1.00	1.00	1.00	526,713	1.00	0.99	0.99	634,080	0.99	1.00	0.99	718,120
ZUPT Points	0.99	1.00	0.99	299,592	0.97	1.00	0.99	304,422	0.99	0.93	0.96	114,572
Avg/Total	1.00	1.00	1.00	826,305	0.99	0.99	0.99	938,502	0.99	0.99	0.99	832,692

**Table 5:** Performance comparisons of the SoC platform and the CPU/GPU platform.

Algorithm	INCU		CNN	
Platform	CPU (i7-6700)	SoC	GPU (1050Ti)	SoC
$\bar{t}$ (s/point)	157.2	276.95	104.46	496.5
Power(w)	65	**2.93**	75	**2.91**
EE(points/J)	97.87	1231.50	127.65	691.42
EER	/	**12.58**	/	**5.42**

**Table 6:** Resource usage of the AZUPT implemented device.

Resource	Utilization	Available	Utilization%
LUT	49,091	53,200	92.28
LUTRAM	7452	17,400	42.83
FF	47,791	106,400	44.92
BRAM	65	140	46.43
DSP	208	220	94.55
IO	14	125	11.20
BUFG	5	32	15.63
MMCM	1	4	25.00
PLL	1	4	25.00

## Data Availability

The study did not report any data.
